# CAREGIVING THROUGH COVID: POSITIVE ASPECTS OF CAREGIVING AND CAREGIVER STRESS OVER TIME

**DOI:** 10.1093/geroni/igad104.2591

**Published:** 2023-12-21

**Authors:** Haley Schneider, Maria Donohoe, Lena Thompson, Sato Ashida

**Affiliations:** University of Iowa, Iowa City, Iowa, United States; University of Iowa College of Public Health, Iowa City, Iowa, United States; University of Minnesota, Duluth, Minnesota, United States; University of Iowa College of Public Health, Iowa City, Iowa, United States

## Abstract

With an increase of age-related diseases due to population-aging, there are more caregivers in need of support services. Caregivers are known to experience poor health status due to caregiving burden, stress, and psychological impacts. Caregivers often experience care-related negative health consequences, and such experiences can be exacerbated during stressful times such as COVID-19 pandemic. The concepts of positive aspects of caregiving (PAC) help identify protective factors (sense of meaningfulness in role, familial cohesion, etc.) that have been shown to decrease negative health impacts and enhance caregiver satisfaction. This study included 43 caregivers of individuals diagnosed with Alzheimer’s Disease or related dementias (ADRDs) who participated in a randomized-controlled trial (2020-2021) to test an intervention that provides caregiver education and referral to community-based services. A regression model was built using the data from baseline and three-month post-intervention surveys. Controlling for baseline COVID-19 distress levels and gender, an increase in COVID-19 distress from baseline to three months was significantly associated with lower levels of PAC at baseline (b=-0.337, p-value=0.029); there was also a trend for a decrease in PAC over three months (b=-0.300, p-value=0.058). Understanding how lower levels of PAC leads caregivers to experience higher stress levels during the pandemic likely will help identify the opportunity to address caregiver stress. Future studies may explore the strategies to enhance caregiver PAC and investigate how changes in PAC may impact caregiver wellbeing. Future interventions may benefit from incorporating the concepts of PAC to support and empower caregivers during stressful events like the pandemic.

